# The lncRNA *UBE2R2-AS1* suppresses cervical cancer cell growth *in vitro*

**DOI:** 10.1515/med-2020-0241

**Published:** 2020-11-29

**Authors:** Chunyan Liu, Huajun Li, Qinan Yin

**Affiliations:** Department of Obstetrics and Gynaecology, China-Japan Friendship Hospital, Beijing, 100029, China; Immunohematology Laboratory, Clinical Center of National Institutes of Health, Bethesda, Maryland, 20852, USA

**Keywords:** *UBE2R2-AS1*, cervical cancer, biological activities, HeLa, SiHa

## Abstract

**Purpose:**

This study explores the effects and mechanisms of the long noncoding RNA (lncRNA) *UBE2R2-AS1* activity in the cervical cancer development.

**Methods:**

Thirty-four pairs of normal adjacent and cancer tissues were collected from cervical cancer patients. Pathology was evaluated by HE staining, and *UBE2R2-AS1* expression was evaluated by *in situ* hybridization assays. HeLa and SiHa cells were respectively divided into negative control, pcDNA 3.1 vehicle control and lncRNA-expressing groups. Cell proliferation and apoptosis were measured by CCK8 expression and flow cytometry. The number of invading cells and the wound healing rate were measured by transwell and wound healing assays, respectively. Relative protein levels (caspase-3, caspase-8, MMP-2 and MMP-9) were measured by Western blot.

**Results:**

Compared with adjacent normal tissues, *UBE2R2-AS1* expression was significantly suppressed in cancer tissues correlated with the increasing stage. *UBE2R2-AS1* suppressed cell proliferation and enhanced apoptosis, as well as decreased cell invasion and wound healing in cervical cancer cell lines. *UBE2R2-AS1* overexpression significantly upregulated caspase-3 and caspase-8 protein expressions and significantly downregulated MMP-2 and MMP-9 protein expressions by Western blot.

**Conclusion:**

*UBE2R2-AS1* suppressed cervical cancer cell biological activities and might represent an antitumor factor in cervical cancer.

## 1 Introduction

Cervical cancer has become the second most common malignant tumor after breast cancer and the third leading cause of cancer death in women worldwide [1,2,3]. The prophylactic human papillomavirus vaccine is important for the prevention of cervical cancer; however, the late relapse of cervical cancer cannot be easily treated, resulting in the high mortality rate, and the pathogenesis of cervical cancer remains unclear. Therefore, there is an urgent need to explore the molecular mechanism of cervical cancer cell invasion and metastasis and to find effective molecular therapeutic targets for diagnosis and treatment.

Long noncoding RNAs (lncRNAs; nontranslated RNA of more than 200 nucleotides) are involved in biological functions primarily through epigenetic, transcriptional, and posttranscriptional regulation of gene expression. lncRNAs with protein-coding capabilities have also recently been identified [4,5,6]. The latest evidence suggests that lncRNAs have essential biological functions closely related to human cancers [7,8]. A number of lncRNAs closely related to cancer have been identified to function in the regulation of pathogenic genes of cervical cancer, such as HOTAIR, H19, GAS5, and MEG3 [9,10,11,12]. These represent important molecular targets, as well as a theoretical basis for the research of the pathogenesis of cervical cancer and early diagnosis and treatment. A recent study has shown that the lncRNA *UBE2R2-AS1*, a tumor suppressor gene, is abnormally expressed in neuroglioma [13]. However, the expression and the role of the lncRNA *UBE2R2-AS1* in cervical cancer are unclear. This study focuses on lncRNA *UBE2R2-AS1* expression in cervical cancer cells and its effect on the biological activities of cells.

## 2 Materials and Methods

### 2.1 Clinical Samples

Thirty-four pairs of cervical tumor and adjacent tissues of patients who had been diagnosed with cervical cancer and undergone radical resections at the Department of Obstetrics and Gynecology of the China–Japan Friendship Hospital from 2016 to June 2018 were obtained. After resection, the tissues were immediately stored in liquid nitrogen for subsequent experiments. All enrolled patients signed the informed consent form and agreed to the use of the specimens in the present study. No patient received radiotherapy or chemotherapy before enrolment, and there was no history of major organ dysfunctionality. Samples were fixed in 4% paraformaldehyde for 24 h and embedded in paraffin. The ethics committee of Sino Japanese Friendship Hospital approved the present study, and all patients or their families signed informed consent.

### 2.2 Pathological H&E staining

Embedded paraffin tissues were sectioned as 4 µm followed by dewaxing, washing off residual xylene, hematoxylin staining, bluing and color separation, eosin staining, dehydration, transparency, and sealing. The stained sections were observed under optical microscopy.

### 2.3 *In situ* hybridization (ISH) staining

Samples were sectioned and dewaxed with xylene. After residual xylene was removed with 100% ethanol, sections were baked at 45–50°C℃ for 2–5 min and washed in 2× SSC buffer for 3 min. Sections were incubated in 1 M sodium cyanide isosulfate (NaSN) at 80°C for 30 min and washed in 2× SSC buffer for 3 min. Samples were placed in 70% formamide/2× SSC solution preheated to 75°C for denaturation for 7 min. Afterward, they were immediately placed in 70, 85, and 100% ethanol precooled to −20°C for gradient dehydration and dried naturally. The probe was incubated at 75°C for denaturation for 7 min, and the samples were maintained at 48°C for hybridization. The dried sections were preheated to 48°C for 5 min. A 10 µL probe (Boster Biological Technology, Wuhan, China) was gently applied onto the tissue samples, and the samples were immediately covered with a coverslip and a sealing film. Samples were placed in a wet box at 43°C overnight for hybridization. After hybridization, the sealing film and the coverslip were removed, and the samples were preheated to 48°C. Then, they were washed in 50% formamide/2× SSC solution three times for 5 min each, washed in 2× SSC buffer for 10 min, washed in 0.11% NP-40/2× SSC solution for 5 min, washed in 75% solution at room temperature for 3 min, and dried in the dark. A total of 15 µL DAP 1 was applied, and then, the sections were sealed and placed in the dark for 20 min. The expression levels of the lncRNA *UBE2R2-AS1* were analyzed using Image J software.

### 2.4 Cell lines and culture

Human cervical cancer cell lines HeLa and SiHa were purchased from the cell bank of the Chinese Academy of Sciences (Shanghai). All cells were cultured with 10% heat-inactivated fetal bovine serum, 100 IU/mL streptomycin, and 100 µg/mL penicillin in Dulbecco’s modified Eagle’s medium (DMEM) and incubated in humidified air containing 5% CO_2_ at 37°C.

### 2.5 Cell culture and transfection

HeLa and SiHa cells were incubated in six-well plates and cultured in RPMI1640 medium containing 10% fetal bovine serum in a cell culture incubator containing 5% CO_2_ at 37°C. Cells were split into a 25 mm^2^ culture dish. Once cells reached 80% confluence, cells were transfected with pcDNA 3.1 (Invitrogen, Carlsbad, CA) using a transfection reagent (Roche, Germany) and harvested 48 h later. The lncRNA *UBE2R2-AS1* sequence was constructed by Nanjing KeyGen Biotech. RT-PCR assay was used to determine the expression level of the lncRNA *UBE2R2-AS1* in each experimental group.

### 2.6 qRT-PCR

Total cellular RNAs were extracted using RNeasy Mimi Kit (QIAGEN). cDNA was synthesized using cDNA Reverse Transcription Kit (Invitrogen, Thermo Fisher Scientific). Quantitative real-time PCR (qRT-PCR) was conducted on a QS6 Fast Real-Time PCR system (Thermo Fisher Scientific) using SYBR Green Master Mix or TaqMan Master Mix (Invitrogen, Thermo Fisher Scientific). The relative expression of lncRNA UBE2R-AS1 was calculated by the 2^−ΔΔCt^ method. Primer sequences used are presented in Table 1.

**Table 1:** The primes sequence in this study

Name	F&R primes	Prime sequences from 5′–3′	Size
UBE2R2-AS1	F	GTCTGGGTAGTCAGCTGTGAGG	129 bp
	R	TCTCCAGAGGCAGTGTTCCTC	
GAPDH	F	AGCCACATCGCTCAGACAC	197 bp
	R	GCCCAATACGACCAAATCC	

### 2.7 CCK-8 assay for cell proliferation

Cells were transfected in a six-well culture plate (5 × 10^3^ cells per well) for 48 h. Each experimental group had three replicates. Ten microliters of CCK-8 solution were added, and after 2 h of incubation, the absorbance value (optical density) of each well was measured by a microplate reader at 450 nm wavelength. Cell proliferation activities were recorded and calculated.

### 2.8 Flow cytometry for apoptosis

Forty-eight hours after transfection, cells were trypsinized and collected in a 15 mL centrifuge tube and centrifuged at 800 × *g* for 3 min. A cell suspension was prepared with 1 mL of phosphate-buffered saline solution. Samples were suspended in 200 µL of binding buffer, and then, 5 µL of Annexin V-FITC and propidium iodide (PI) staining solution were added to each tube. The samples were tested using a FACSAria Type II flow cytometer (BD, USA), and the apoptotic rate was recorded.

### 2.9 Transwell chamber assay for cell invasion

Forty-eight hours after transfection, cells were harvested, and the cell concentration was adjusted to 5 × 10^5^ cells/mL with serum-free medium. Three hundred microliters of serum-free medium was added to the transwell chamber. Then, 500 µL of medium containing 10% serum and 300 µL of single-cell suspensions were added to the lower chamber of transwell. After incubation for 24 h, 500 µL of 0.1% solution was used to stain the cells, which were then counted via microscopy, and the cell invasion rate was recorded.

### 2.10 Wound healing test for cell migration

Forty-eight hours after transfection, when cell density reached 60%, a 10 µL white pipette tip was used to gently draw three to five equidistant parallel lines at the bottom of the dish. The cells were photographed 24 and 48 h after wounding. The scratch area at each time point was calculated, and the wound healing rate of the cells was recorded.

### 2.11 Western blot assay

RIPA buffer was used to lyse cells, and the total protein concentration was quantified using the BCA method. The samples were loaded at 30 µg per well and transferred to the PVDF membrane following electrophoresis. After blocking, rabbit anti-human caspase-3 (1:1,000), caspase-8 (1:1,000), MMP-2 (1:1,000), MMP-9 (1:1,000), and GAPDH (1:500) were added, and the samples were incubated at 37°C for 2 h. After washing, horseradish peroxidase-labeled goat antirabbit and goat antimouse IgG were added. Images were obtained using an ECL gel imaging system to obtain the target protein expression band.

### 2.12 Statistical analysis

Statistical analysis was conducted using IBM SPSS Statistics Version 21.00 software. Measurement data were expressed as means ± standard deviation (means ± SD). Two independent samples or multiple sets of samples were analyzed by *t*-test or one-way ANOVA with a significance level of *P* ≤ 0.05.

## 3 Results

### 3.1 Clinical pathology and lncRNA UBE2R2-AS1 expression

**Figure 1:**
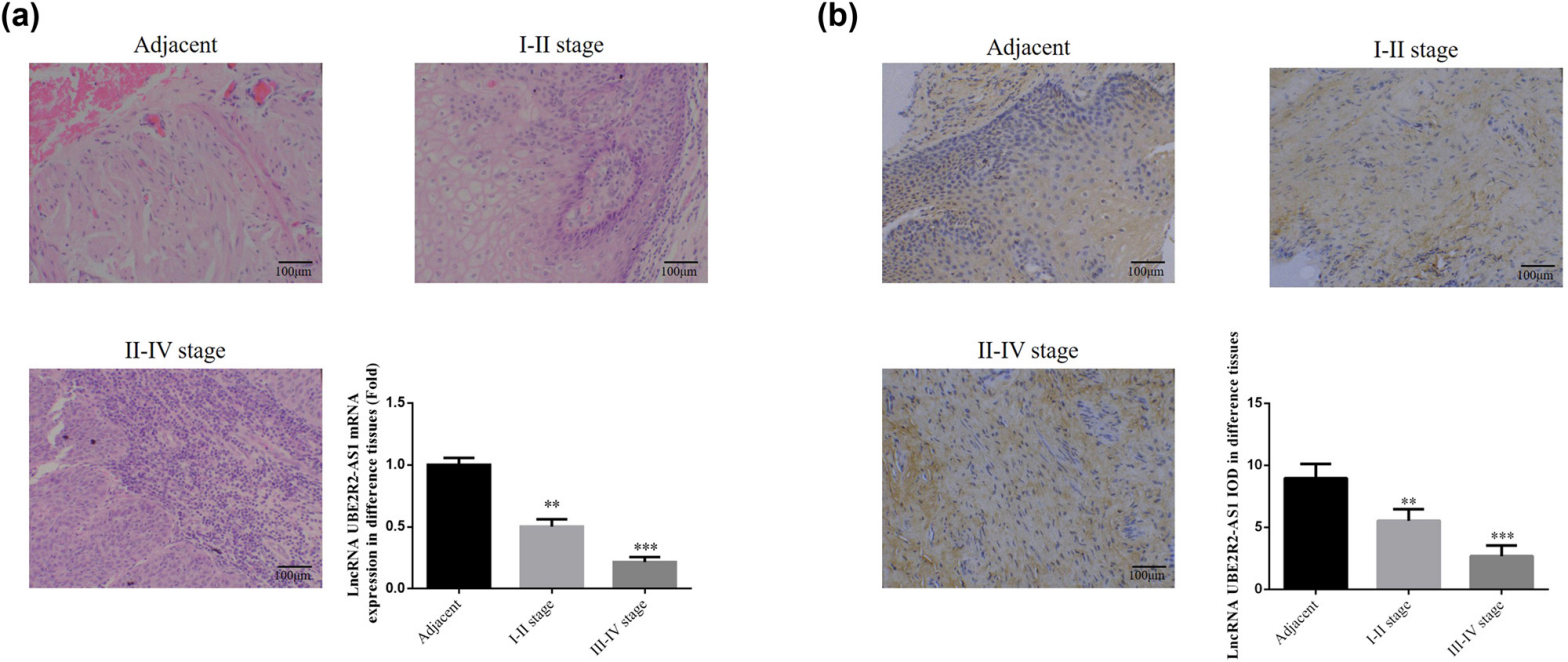
Clinical pathology and lncRNA UBE2R2-AS1 expression. Adjacent: adjacent normal tissues; I–II stage: I–II stage cervical cancer tissues; III–IV stage: III–IV stage cervical cancer tissues. (a) Clinical pathology by HE staining (200×) and lncRNA UBE2R2-AS1 mRNA expression. **P < 0.01 and ***P < 0.001, compared with NC group. (b) lncRNA UBE2R2-AS1 expression by ISH assay (200×) **P < 0.01, ***P < 0.001, compared with NC group.

Cell invasion and migration were aggravated in cervical cancer tissues positively correlated with the increasing stage (Figure 1a). Compared with adjacent normal tissues, lncRNA *UBE2R2-AS1* expression in cervical cancer tissues was significantly decreased with the increasing stage (*P* < 0.01, Figure 1b).

#### 3.1.1 Cell viability in HeLa and SiHa cell lines

**Figure 2:**
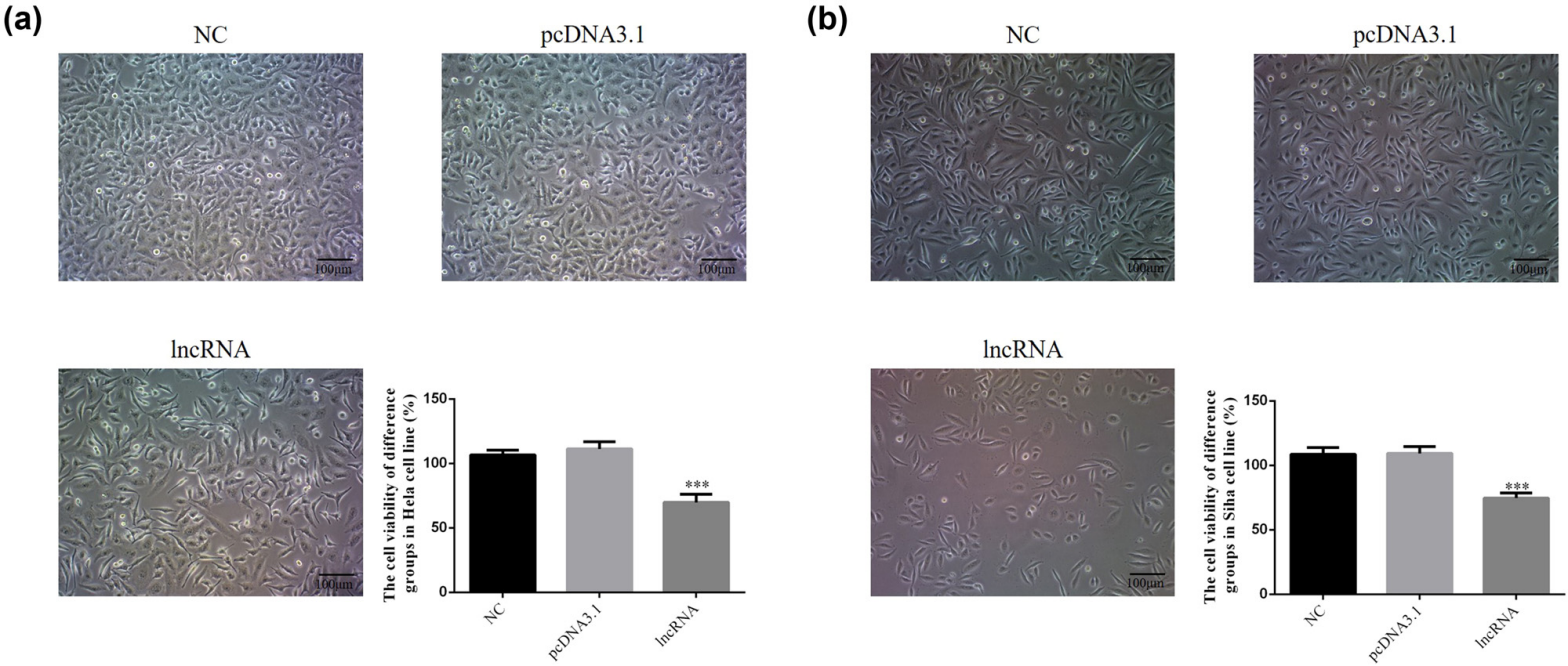
The cell viability of difference groups by CCK-8 in HeLa and SiHa cell lines. NC: normal control group; pcDNA 3.1: the cells were transfected with pcDNA3.1; lncRNA: the cells were transfected with UBE2R2-AS1 by pcDNA3.1. (a) The cell viability of difference groups by CCK-8 in HeLa cell. ***P < 0.001, compared with NC group. (b) The cell viability of difference groups by CCK-8 in SiHa cell. ***P < 0.001, compared with NC group.

Cell morphology in HeLa and SiHa cell lines was not significantly different among treatment groups in HeLa and SiHa cells. These results indicate that *UBE2R2-AS1* transfection did not affect cell morphology. Compared with the negative control (NC) group, the cell viability of lncRNA-transfected groups was significantly decreased in HeLa and SiHa cell lines (Figure 2a and b).

#### 3.1.2 The cell apoptosis by flow cytometry assay

**Figure 3:**
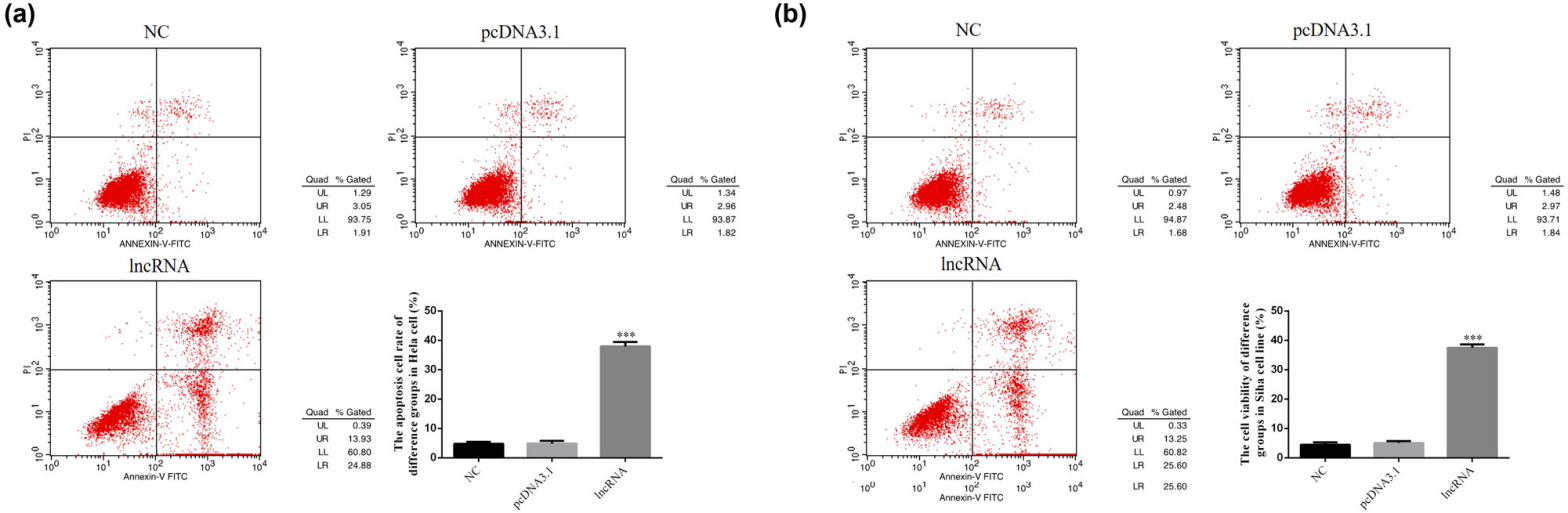
The cell apoptosis rate of difference groups by flow cytometry in HeLa and SiHa cell lines. NC: normal control group; pcDNA 3.1: the cells were transfected with pcDNA3.1; lncRNA: the cells were transfected with UBE2R2-AS1 by pcDNA3.1. (a) The cell apoptosis rate of difference groups by flow cytometry in HeLa cell. ***P < 0.001, compared with NC group. (b) The cell apoptosis rate of difference groups by flow cytometry in SiHa cell. ***P < 0.001, compared with NC group.

The rate of cellular apoptosis in lncRNA-transfected groups was significantly increased compared with those of the NC group (*P* < 0.001, Figure 3a and b); meanwhile, there were no significant differences between the NC and pcDNA 3.1 vehicle-transfected groups in HeLa and SiHa cell lines.

#### 3.1.3 The lncRNA UBE2R2-AS1 affects cell invasion in HeLa and SiHa cell lines

**Figure 4:**
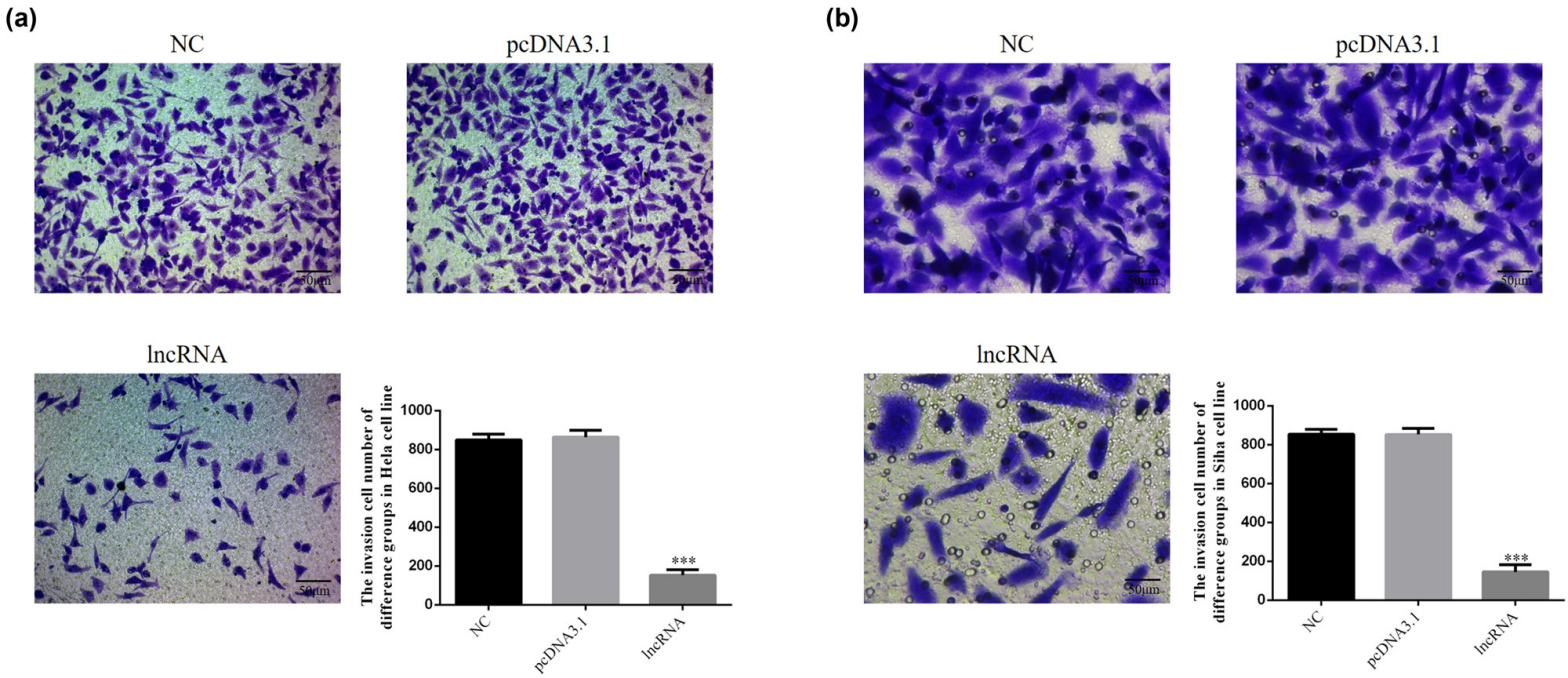
UBE2R2-AS1 affected invasion cell number of difference groups in HeLa and SiHa cell lines (200×). NC: normal control group; pcDNA 3.1: the cells were transfected with pcDNA3.1; lncRNA: the cells were transfected with UBE2R2-AS1 by pcDNA3.1. (a) UBE2R2-AS1 affected invasion cell number of difference groups in HeLa cell. ***P < 0.001, compared with NC group. (b) UBE2R2-AS1 affected invasion cell number of difference groups in SiHa cell. ***P < 0.001, compared with NC group.

Compared with the NC group, the number of invading cells in the lncRNA-transfected groups was significantly lower (*P* < 0.001, Figure 4a and b). There were no significant differences between the NC and pcDNA 3.1 vehicle-transfected groups in HeLa and SiHa cell lines.

#### 3.1.4 The lncRNA UBE2R2-AS1 affects cell migration in HeLa and SiHa cell lines

**Figure 5:**
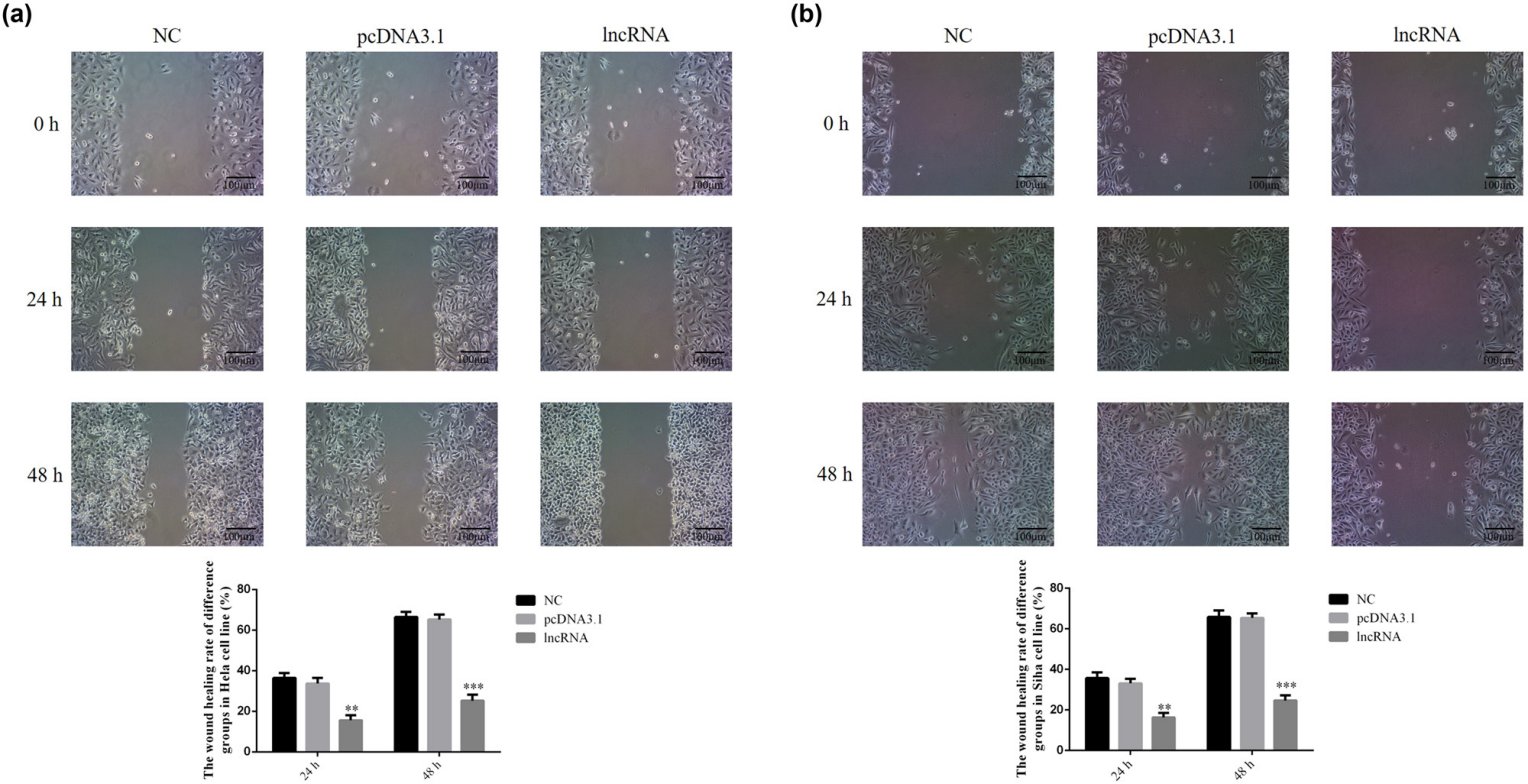
UBE2R2-AS1 affected the wound healing rate of difference groups in HeLa and SiHa cell lines by wound healing assay (100×). NC: normal control group; pcDNA 3.1: the cells were transfected with pcDNA3.1; lncRNA: the cells were transfected with UBE2R2-AS1 by pcDNA3.1. (a) UBE2R2-AS1 affected wound healing rate of difference groups in HeLa cell. ***P < 0.001, compared with NC group. (b) UBE2R2-AS1 affected wound healing rate of difference groups in SiHa cell. ***P < 0.001, compared with NC group.

Compared with the NC group, the wound healing rate of lncRNA-transfected groups was significantly decreased (*P* < 0.001, Figure 5a and b) after 24 and 48 h. There were no significant differences between the NC and pcDNA 3.1 vehicle-transfected groups in HeLa and SiHa cell lines.

#### 3.1.5 The lncRNA UBE2R2-AS1 affects relative protein expressions by WB assay

**Figure 6:**
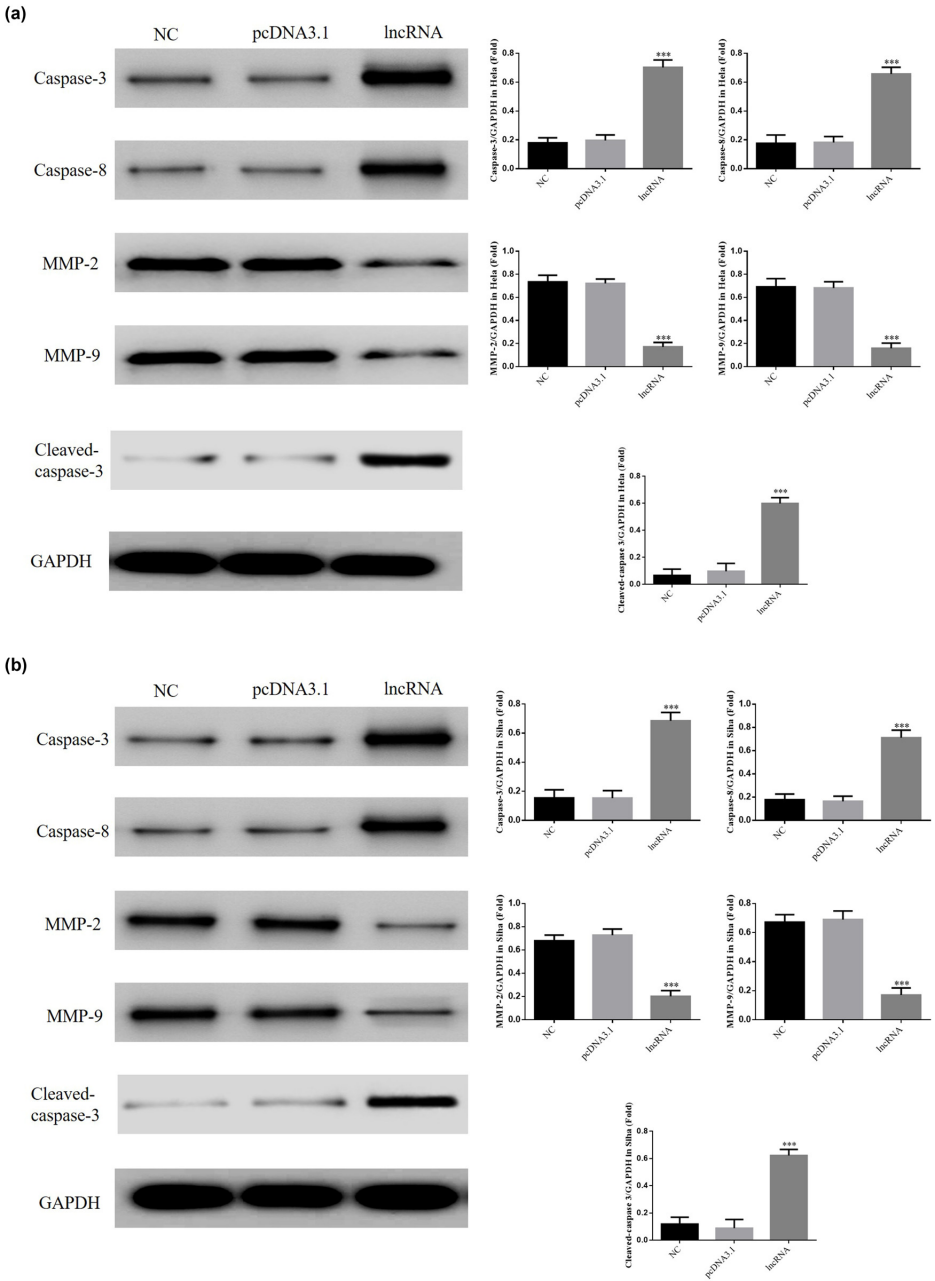
UBE2R2-AS1 affected relative proteins expressions by WB assay. NC: normal control group; pcDNA 3.1: the cells were transfected with pcDNA3.1; lncRNA: the cells were transfected with UBE2R2-AS1 by pcDNA3.1. (a) UBE2R2-AS1 affected relative proteins expressions by WB assay in HeLa cell. ***P < 0.001, compared with NC group. (b) UBE2R2-AS1 affected relative proteins expressions by WB assay in SiHa cell. ***P < 0.001, compared with NC group.

Compared with the NC group, expressions of caspase-3 and caspase-8 proteins were significantly upregulated, and expressions of MMP-2 and MMP-9 proteins were significantly downregulated in lncRNA-transfected HeLa and SiHa cell lines (*P* < 0.001, Figure 6a and b). There were no significant differences between the NC and pcDNA 3.1 vehicle-transfected groups in HeLa and SiHa cells.

## 4 Discussion

Cervical cancer is one of the most common types of malignant tumor in middle-aged and older women. The pathogenesis of this disease remains unclear, and the existing treatment methods are largely ineffective. Invasion and metastasis of cervical cancer cells are the main causes of death in patients with cervical tumors. Therefore, there is an urgent need to explore the mechanisms of invasion and metastasis of tumor cells and to seek new therapeutic targets and strategies. This could provide new ideas for reducing the mortality of patients with cervical tumors.

lncRNAs have been a research hotspot in recent years. A series of studies have demonstrated that the expression of lncRNAs is closely related to the occurrence and the development of tumors. Unlike mRNAs that code proteins, most lncRNAs are expressed in an extremely tissue-specific manner. Yang et al. found that the expression of the lncRNA *PVT1* is abnormally elevated in the serum of patients with cervical cancer. This closely correlated with the tumor size and the clinical stage of patients and could be used as a molecular marker for the early diagnosis of cervical cancer [14]. Cao et al. found that the expression level of the lncRNA *SPRY4-IT1* in cervical cancer tissues was higher than that in adjacent tissues. This could be used as an independent molecular marker to determine the survival and the prognosis of patients with cervical cancer [15]. lncRNAs play an important role in the occurrence and the development of cervical cancer; however, only a small number of lncRNAs have been well studied, whereas the expression and functions of most lncRNAs have not been fully investigated.

The lncRNA *UBE2R2-AS1* is a newly discovered lncRNA [13,16]. In the present study, tumor and adjacent normal tissues were collected from patients with cervical cancer, and ISH was used to examine the difference in lncRNA *UBE2R2-AS1* expression. These results showed that *UBE2R2-AS1* expression was significantly lower in cervical cancer tissues. In subsequent cell experiments, *UBE2R2-AS1* was transfected into HeLa and SiHa cells, and proliferation, invasion, and migration of cervical cancer cell lines HeLa and SiHa were significantly inhibited. To further explore the specific mechanism of *UBE2R2-AS1* in cervical cancer, the expression of related proteins was investigated.

In the present study, flow cytometry confirmed that after *UBE2R2-AS1* was transfected into HeLa and SiHa cells, apoptosis was evident. A key step in inducing apoptosis is the activation of caspase-3, which is inseparable from the activation of upstream factor caspase-8. The activation of caspase-3 represents the point of no return in apoptosis [17]. Western blot assays showed that the activities of caspase-3 and caspase-8 were significantly increased following overexpression of *UBE2R2-AS1*. These caspases can execute caspase cascades and induce apoptosis by regulating the release of cytochrome c in mitochondria [18,19]. *UBE2R2-AS1* overexpression leads to the activation of caspase-3 and caspase-8, which may be the cause of increased apoptosis in HeLa and SiHa cells and thus inhibit cell proliferation.

MMPs are calcium-dependent zinc-containing endogenous peptidases that degrade various ECM proteins [20,21]. One of the main significance of MMPs in cancer stress is this role in ECM degradation. Together with MMP-9, MMP-2 can degrade the most abundant component of the basement membrane, type IV collagen. Degradation of the ECM causes cancer cells to metastasize from primary tumors [22,23,24]. In the present study, following transfection of *UBE2R2-AS1* into HeLa and SiHa cells, the invasion and migration of cervical cancer cell lines were significantly inhibited. WB assays showed that the expression levels of MMP-2 and MMP-9 proteins were significantly reduced, indicating that *UBE2R2-AS1* inhibits the invasion and the migration of cervical cancer cells by regulating MMP-2/9 expression.

In conclusion, UBE2R2-AS1 is an important tumor suppressor gene in cervical cancer, and its expression level correlates with the occurrence of cervical cancer. UBE2R2-AS1 is a potential therapeutic target and a prognostic biomarker for cervical cancer and may be used for the monitoring of cervical cancer. This finding provides a new molecular target for future research on the invasion and progression of cervical cancer.

## References

[1] Thorsteinsson K, Ladelund S, Jensen-Fangel S, Katzenstein TL, Johansen IS, Pedersen G, et al. Incidence of cervical dysplasia and cervical cancer in women living with HIV in Denmark: comparison with the general population. HIV Med. 2016 Jan;17(1):7–17. 10.1111/hiv.12271 26058995

[2] Hoque ME, Ghuman S, Coopoosmay R, Van, Hal G. Cervical cancer screening among university students in South Africa: a theory based study. PLoS One. 2014 Nov 11;9(11):e111557. 10.1371/journal.pone.0111557 PMC4227671 25387105

[3] Mathieu EL, Belhocine M, Dao LT, Puthier D, Spicuglia S. Functions of lncRNA in development and diseases. Med Sci (Paris). 2014 Aug–Sep;30(8-9):790–6. 10.1051/medsci/20143008018 25174757

[4] Peng L, Yuan X, Jiang B, Tang Z, Li GC. LncRNAs: key players and novel insights into cervical cancer. Tumour Biol. 2016 Mar;37(3):2779–88. 10.1007/s13277-015-4663-9 26715267

[5] Kornienko AE, Guenzl PM, Barlow DP, Pauler FM. Gene regulation by the act of long non-coding RNA transcription. BMC Biol. 2013 May 30;11:59. 10.1186/1741-7007-11-59 PMC3668284 23721193

[6] Xu XF, Li J, Cao YX, Chen DW, Zhang ZG, He XJ, et al. Differential expression of long noncoding RNAs in human cumulus cells related to embryo developmental potential: a microarray analysis. Reprod Sci. 2015 Jun;22(6):672–8. 10.1177/1933719114561562 PMC4502809 25527423

[7] Chalei V, Sansom SN, Kong L, Lee S, Montiel JF, Vance KW, et al. The long non-codign RNA Dali is an epigenetic regulator of neural differentiation. Elife. 2014 Nov 21;3:e04530. 10.7554/eLife.04530 PMC4383022 25415054

[8] Taft RJ, Pang KC, Mercer TR, Dinger M, Mattick JS. Non-coding RNAs: regulation of disease. J Pathol. 2010 Jan;220(2):126–39. 10.1002/path.2638 19882673

[9] Qin R, Chen Z, Ding Y, Hao J, Hu J, Guo F. Long non-coding RAN MEG3 inhibits the proliferation of cervical carcinoma cells through the induction of cell cycle arrest and apoptosis. Neoplasma. 2013;60(5):486–92. 10.4149/neo_2013_063 23790166

[10] Huang L, Liao LM, Liu AW, Wu JB, Cheng XL, Lin JX, et al. Overexpression of long noncoding RNA HOTAIR predicts a poor prognosis in patients with cervical cancer. Arch Gynecol Obstet. 2014 Oct;290(4):717–23. 10.1007/s00404-014-3236-2 24748337

[11] Cao S, Liu W, Li F, Zhao W, Qin C. Decreased expression of lncRNA GASS predicts a poor prognosis in cervical cancer. Int J Clin Exp Pathol. 2014 Sep 15;7(10):6776–83. PMC4230116 25400758

[12] Kim SJ, Park SE, Lee C, Lee SY, Jo JH, Kim JM, et al. Alterations in promoter usage and expression levels of insulin-like growth factor-II and H19 genes in cervical carcinoma exhibiting biallelic expression of IGF-II. Biochim Biophys Acta. 2002 Apr 24;1586(3):307–15. 10.1016/s0925-4439(01)00109-0 11997082

[13] Xu W, Hu GQ, Da Costa C, Tang JH, Li QR, Du L, et al. Long noncoding RNA UBE2R2-AS1 promotes glioma cell apoptosis via targeting the miR-877-3p/TLR4 axis. Onco Targets Ther. 2019 May 7;12:3467–80. 10.2147/OTT.S201732 PMC6511244 31123407

[14] Yang JP, Yang XJ, Xiao L, Wang Y. Long noncoding RNA PVT1 as a novel serum biomarker for detection of cervical cancer. Eur Rev Med Pharmacol Sci. 2016 Oct;20(19):3980–6. 27775803

[15] Cao Y, Liu Y, Lu X, Wang Y, Qiao H, Liu M. Upregulation of long noncoding RNA SPRY4-IT1 correlates with tumor progression and poor prognosis in cervical cancer. FEBS Open Bio. 2016 Aug 9;6(9):954–60. 10.1002/2211-5463.12102 PMC5011494 27642559

[16] Zhou M, Zhang Z, Zhao H, Bao S, Cheng L, Sun J. An immune-related six-lncRNA signature to improve prognosis prediction of glioblastoma multiforme. Mol Neurobiol. 2018 May;55(5):3684–97. 10.1007/s12035-017-0572-9 28527107

[17] Liu Y, Chen H, Zhang L, Zhang T, Ren X. The association between thyroid injury and apoptosis, and alterations of Bax, Bcl-2, and caspase-3 mRNA/protein expression induced by nickel sulfate in wistar rats. Biol Trace Elem Res. 2020;195(1):159–68. 10.1007/s12011-019-01825-0 31392545

[18] Yang JH, Chou CC, Cheng YW, Sheen LY, Chou MC, Yu HS, et al. Effects of glycolic acid on the induction of apoptosis via caspase-3 activation in human leukemia cell line (HL-60). Food Chem Toxicol. 2004 Nov;42(11):1777–84. 10.1016/j.fct.2004.07.004 15350675

[19] Ikner A, Ashkenazi A. TWEAK induces apoptosis through a death-signaling complex comprising receptor-interacting protein 1 (RIP1), Fas-associated death domain (FADD), and caspase-8. J Biol Chem. 2011 Jun 17;286(24):21546–54. 10.1074/jbc.M110.203745 PMC3122213 21525013

[20] Hamsa TP, Kuttan G. Berberine inhibits pulmonary metastasis through down-regulation of MMP in metastatic B16F-10 melanoma cells. Phytother Res. 2012 Apr;26(4):568–78. 10.1002/ptr.3586 21953764

[21] Dab H, Hachani R, Hodroj W, Sakly M, Bricca G, Kacem K. Interaction between sympathetic nervous system and renin angiotensin system on MMPs expression in juvenile rat aorta. Gen Physiol Biophys. 2011 Sep;30(3):271–7. 10.4149/gpb_2011_03_271 21952436

[22] Saladi SV, Keenen B, Marathe HG, Qi H, Chin KV, de la Serna IL. Modulation of extracellular matrix/adhesion molecule expression by BRG1 is associated with increased melanoma invasiveness. Mol Cancer. 2010 Oct 22;9:280. 10.1186/1476-4598-9-280 PMC3098014 20969766

[23] Ming Y, Chen Z, Chen L, Lin D, Tong Q, Zheng Z, et al. Ginsenoside compound K attenuates metastatic growth of hepatocellular carcinoma, which is associated with the translocation of nuclear lacotr-kappaB p65 and reducetion of matrix metalloproteinase-2/9. Planta Med. 2011 Mar;77(5):428–33. 10.1055/s-0030-1250454 20979019

[24] Planagumà J, Liljeström M, Alameda F, Bützow R, Virtanen I, Reventós J, et al. Matrix metalloproteinase-2 and matrix metalloproteinase-9 codistribute with transcription factors RUNX1/AML1 and ETV5/ERM at the invasive front of endometrial and ovarian carcinoma. Hum Pathol. 2011 Jan;42(1):57–67. 10.1016/j.humpath.2010.01.025 20970160

